# *catena*-Poly[[[*N*′-(4-cyano­benzyl­idene)nicotinohydrazide)silver(I)]-μ-*N*′-(4-cyano­benzyl­idene)nicotinohydrazide] hexa­fluorido­phosphate]

**DOI:** 10.1107/S1600536808038907

**Published:** 2008-11-26

**Authors:** Xin-Sheng Wan, Yu-Li Dang, Chun-Hong Kou, Zhan-Fang Zhou, Cao-Yuan Niu

**Affiliations:** a College of Sciences, Henan Agricultural University, Zhengzhou 450002, People’s Republic of China

## Abstract

In the title polymer, {[Ag(C_14_H_10_N_4_O)_2_]PF_6_}_*n*_, each Ag^I^ ion is coordinated by two N atoms from two pyridyl rings of independent *N*′-(4-cyano­benzyl­idene)nicotinohydrazide ligands, and one N atom from one carbonitrile group of a symmetry-related ligand in a distorted T-shaped geometry. The ligands exhibit two modes of coordination. One acts as a bridge connecting Ag atoms to form one-dimensional chains along [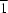
01]. The other acts as a terminal monodentate ligand, coordinating to Ag through its pyridyl N atom. Two neighbouring anti­parallel chains in the crystal are connected through N—H⋯O hydrogen bonds. Other adjacent chains are packed *via* Ag⋯O inter­actions, with Ag⋯O separations of 2.876 (2) Å. In addition, PF_6_^−^ counter-anions inter­act with the hydrazone groups through N—H⋯F hydrogen bonds. The PF_6_^−^ anion is disordered over two sites, with occupancies of 0.773 (8) and 0.227 (8).

## Related literature

For background on fluorescent silver coordination complexes, see: Dong *et al.* (2004 ▶); Sumby & Hardie (2005 ▶). For related structures, see: Niu *et al.* (2007 ▶, 2008 ▶); Vatsadze *et al.* (2004 ▶); Zheng *et al.* (2003 ▶).
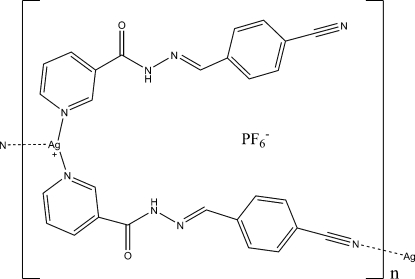


## Experimental

### 

#### Crystal data

[Ag(C_14_H_10_N_4_O)_2_]PF_6_
*M* *_r_* = 753.36
Monoclinic, 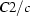

*a* = 22.3252 (17) Å
*b* = 13.6939 (11) Å
*c* = 19.8523 (16) Å
β = 99.9770 (10)°
*V* = 5977.4 (8) Å^3^
*Z* = 8
Mo *K*α radiation
μ = 0.81 mm^−1^
*T* = 173 (2) K
0.44 × 0.32 × 0.29 mm

#### Data collection

Siemens SMART CCD area-detector diffractometer
Absorption correction: multi-scan (*SADABS*; Siemens, 1996 ▶) *T*_min_ = 0.718, *T*_max_ = 0.799
19020 measured reflections
6823 independent reflections
5105 reflections with *I* > 2σ(*I*)
*R*_int_ = 0.021

#### Refinement

*R*[*F* ^2^ > 2σ(*F* ^2^)] = 0.037
*wR*(*F* ^2^) = 0.099
*S* = 1.03
6823 reflections
460 parameters
96 restraints
H atoms treated by a mixture of independent and constrained refinement
Δρ_max_ = 0.92 e Å^−3^
Δρ_min_ = −0.67 e Å^−3^

### 

Data collection: *SMART* (Siemens, 1996 ▶); cell refinement: *SAINT* (Siemens, 1996 ▶); data reduction: *SAINT*; program(s) used to solve structure: *SHELXL97* (Sheldrick, 2008 ▶); program(s) used to refine structure: *SHELXL97* (Sheldrick, 2008 ▶); molecular graphics: *SHELXL97* and *DIAMOND* (Brandenburg, 2005 ▶); software used to prepare material for publication: *SHELXL97*.

## Supplementary Material

Crystal structure: contains datablocks I, global. DOI: 10.1107/S1600536808038907/bh2203sup1.cif

Structure factors: contains datablocks I. DOI: 10.1107/S1600536808038907/bh2203Isup2.hkl

Additional supplementary materials:  crystallographic information; 3D view; checkCIF report

## Figures and Tables

**Table:** 

Ag1—N1	2.172 (2)
Ag1—N2	2.199 (2)
Ag1—N8^i^	2.456 (3)

**Table:** 

N1—Ag1—N2	156.22 (8)
N1—Ag1—N8^i^	109.53 (9)
N2—Ag1—N8^i^	92.22 (9)

Symmetry code: (i) 
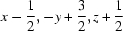
.

**Table 2:** Hydrogen-bond geometry (Å, °)

*D*—H⋯*A*	*D*—H	H⋯*A*	*D*⋯*A*	*D*—H⋯*A*
N3—H28⋯O2^ii^	0.865 (18)	2.15 (2)	2.990 (3)	162 (3)
N6—H29⋯F5	0.853 (18)	2.21 (2)	3.001 (4)	155 (3)

Symmetry code: (ii) 
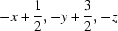
.

**Fig. 1.**
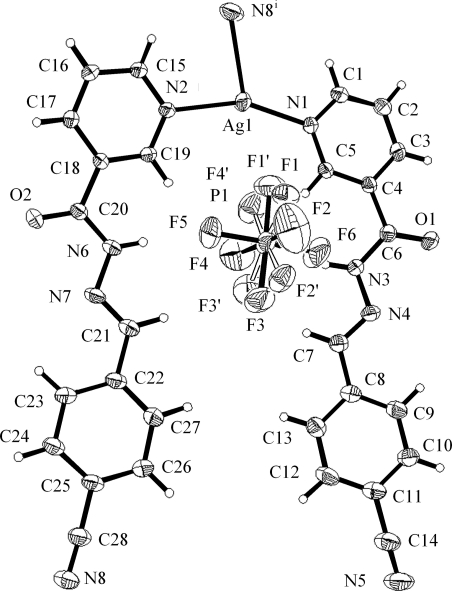
A view of the AgI coordination environment in the polymeric structure of (I), showing the atom-labelling scheme. Displacement ellipsoids are drawn at the 30% probability level and H atoms are shown as small spheres of arbitrary radii [Symmetry code: (i) x - 1/2, -y + 3/2, z + 1/2].

**Fig. 2.**
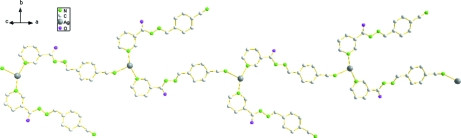
A ball and stick diagram showing the one-dimensional chain. All counteranions and H atoms have been omitted for clarity.

**Fig. 3.**
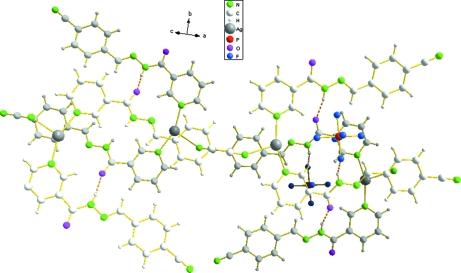
A diagram showing the intermolecular hydrogen bonds, indicated by dashed lines.

## supplementary crystallographic information

### Comment

Pyridyl organic ligands with carbonitrile groups can be used to construct silver coordination complexes with fascinating structures and good fluorescent properties (Sumby & Hardie, 2005; Dong *et al.*, 2004). We also synthesized one-dimensional and two-dimensional silver coordination polymers using this kind of ligands (Niu *et al.*, 2007, 2008). Herein, a one-dimensional silver coordination polymer constructed with a new bridging ligand of this type, 4-cyanobenzylidene nicotinohydrazide, is reported.

In the title compound, (I), the central Ag^I^ ion is coordinated by two N atoms from two pyridyl rings of two different ligands (N1, N2) and one N atom from one carbonitrile group of another ligand [N8^i^, symmetry code: (i) *x* - 1/2, -*y* + 3/2, *z* + 1/2], forming a slightly distorted T-shaped coordination environment (Fig. 1). The N1—Ag1—N2 bond angle is 156.22 (8)°, indicating these three atoms are not exactly in one line. Bond angles N1—Ag1—N8^i^ and N2—Ag1—N8^i^ are larger than 90° (Table 1). The Ag—N bond distances for pyridyl rings are in the range 2.172 (2)–2.199 (2) Å, which is smaller than N—Ag bond distance for the carbonitrile group (Table 1).

One 4-cyanobenzylidene nicotinohydrazide molecule acts as a µ_2_-bridging ligand, by coordinating pyridyl and carbonitrile N atoms. Each bridging ligand connects two silver atoms together by one pyridyl N atom (N1) and one carbonitrile N atom (N8) to form a one-dimensional chain along the [-1 0 1] direction. The separation between two neighbouring Ag atoms in one chain is about 16 Å. Meanwhile, the other independent ligand is acting as a terminal ligand, being coordinated to Ag only through a pyridyl N atom. Two terminal ligands connected to two adjacent Ag atoms in one chain are located at the opposite positions away from the chain (Fig. 2).

There are hydrogen bonds between uncoordinating groups, including pyridyl rings of terminal ligands and all hydrazone groups, as well as other groups like counteranions. On one hand, counteranions PF_6_^-^ interact with the ligands in the polymer through N—H···F hydrogen bonds (Table 2). Four F atoms (F1 to F4) of the PF_6_^-^ anion are disordered over two sites, with occupancies 0.773 (8) and 0.227 (8). On the other hand, there are also N—H···O hydrogen bonds between two neighbouring antiparallel chains in the crystal (Fig. 3). In addition to these intermolecular contacts, there are weak Ag···O interactions between one O atom (O1) of the terminal ligand and one Ag atom in the neighbouring chain, with Ag···O separations of 2.876 (2) Å. These noncovalent interactions have large contributions to the supramolecular three-dimensional framework.

One-dimensional Ag^I^ coordination polymers with T-shaped coordination geometry were previously described in a few compounds: {[Ag(2,6-di(3-pyridylmethylidene) cyclohexanone)](NO_3_)}*_n_* (Vatsadze *et al.*, 2004) and {[Ag(2,2'-(methylenebis(thio)) bis(pyrimidine))](NO_3_)}*_n _*(Zheng *et al.*, 2003). The N—Ag—N bond angles in these two compounds deviate from 180° (*ca*. 158 and 131°, respectively), which are close to that observed in (I).

### Experimental

A solution of AgPF_6_ (0.025 g, 0.1 mmol) in CH_3_OH (10 ml) was carefully layered on a CH_3_OH/CHCl_3_ solution (5 ml/10 ml) of 4-cyanobenzylidene nicotinohydrazide (0.025 g, 0.1 mmol) in a straight glass tube. About ten days later, colourless single crystals suitable for X-ray analysis were obtained (yield: *ca*. 50%). Analysis, calculated for C_28_H_20_AgN_8_O_2_F_6_P: C 44.64, H 2.68, N 14.87%; found: C 44.79, H 2.69, N 14.99%.

### Refinement

C-bound H atoms were placed in calculated positions and refined using a riding model [C—H = 0.95 Å and *U*_iso_(H) = 1.2*U*_eq_(C)]. The N-bound H atoms were first introduced in calculated positions, and then their positions and displacement parameters were refined with the N—H bond lengths restrained to 0.88 (2) Å. Four F atoms (F1/F2/F3/F4) of the hexafluorophosphate anion are disordered over two positions (F1'/F2'/F3'/F4'), and all P—F bond lengths were restrained to a target value of 1.58 (2) Å. Displacement parameters for disordered F atoms were also subjected to restraints. The final difference map had a highest peak at 0.88 Å from Ag1 and a deepest hole at 0.71 Å from Ag1, but was otherwise featureless.

### Crystal data

**Table:**

[Ag(C_14_H_10_N_4_O)_2_]PF_6_	*F*_000_ = 3008
*M**_r_* = 753.36	*D*_x_ = 1.674 Mg m^−^^3^
Monoclinic, *C*2/*c*	Mo *K*α radiation λ = 0.71073 Å
Hall symbol: -C 2yc	Cell parameters from 5745 reflections
*a* = 22.3252 (17) Å	θ = 2.1–27.5º
*b* = 13.6939 (11) Å	µ = 0.81 mm^−^^1^
*c* = 19.8523 (16) Å	*T* = 173 (2) K
β = 99.9770 (10)º	Prism, colourless
*V* = 5977.4 (8) Å^3^	0.44 × 0.32 × 0.29 mm
*Z* = 8	

### Data collection

**Table:**

Siemens SMART CCD area-detector diffractometer	6823 independent reflections
Radiation source: fine-focus sealed tube	5105 reflections with *I* > 2σ(*I*)
Monochromator: graphite	*R*_int_ = 0.022
*T* = 173(2) K	θ_max_ = 27.5º
φ and ω scans	θ_min_ = 2.1º
Absorption correction: multi-scan(SADABS; Siemens, 1996)	*h* = −28→25
*T*_min_ = 0.718, *T*_max_ = 0.800	*k* = −16→17
19020 measured reflections	*l* = −25→25

### Refinement

**Table:**

Refinement on *F*^2^	Secondary atom site location: difference Fourier map
Least-squares matrix: full	Hydrogen site location: inferred from neighbouring sites
*R*[*F*^2^ > 2σ(*F*^2^)] = 0.037	H atoms treated by a mixture of independent and constrained refinement
*wR*(*F*^2^) = 0.099	*w* = 1/[σ^2^(*F*_o_^2^) + (0.0457*P*)^2^ + 5.9377*P*] where *P* = (*F*_o_^2^ + 2*F*_c_^2^)/3
*S* = 1.03	(Δ/σ)_max_ = 0.001
6823 reflections	Δρ_max_ = 0.92 e Å^−^^3^
460 parameters	Δρ_min_ = −0.67 e Å^−^^3^
96 restraints	Extinction correction: none
Primary atom site location: structure-invariant direct methods	

### Fractional atomic coordinates and isotropic or equivalent isotropic displacement parameters (Å^2^)

**Table:**

	*x*	*y*	*z*	*U*_iso_*/*U*_eq_	Occ. (<1)
N8	0.61351 (13)	0.7668 (2)	−0.23514 (14)	0.0680 (8)	
C28	0.57801 (14)	0.7655 (3)	−0.20064 (15)	0.0538 (7)	
C25	0.53188 (13)	0.7592 (2)	−0.15834 (14)	0.0478 (7)	
C24	0.50609 (14)	0.6686 (2)	−0.14987 (15)	0.0531 (7)	
H24	0.5184	0.6128	−0.1724	0.064*	
C26	0.51433 (15)	0.8411 (3)	−0.12646 (17)	0.0601 (8)	
H26	0.5315	0.9031	−0.1330	0.072*	
C27	0.47114 (15)	0.8310 (2)	−0.08462 (18)	0.0601 (8)	
H27	0.4592	0.8868	−0.0618	0.072*	
C23	0.46282 (14)	0.6602 (2)	−0.10881 (15)	0.0510 (7)	
H23	0.4449	0.5985	−0.1032	0.061*	
C22	0.44513 (13)	0.7418 (2)	−0.07544 (14)	0.0477 (7)	
C21	0.40138 (14)	0.7334 (2)	−0.02860 (15)	0.0520 (7)	
H21	0.3960	0.7864	0.0006	0.062*	
N7	0.37061 (11)	0.6556 (2)	−0.02684 (12)	0.0494 (6)	
N6	0.33253 (11)	0.6554 (2)	0.02109 (12)	0.0486 (6)	
Ag1	0.182725 (12)	0.812121 (16)	0.197931 (13)	0.05723 (10)	
C12	0.44862 (16)	1.2299 (3)	−0.09411 (17)	0.0643 (9)	
H12	0.4699	1.1967	−0.1248	0.077*	
C19	0.24057 (13)	0.66761 (18)	0.10608 (13)	0.0401 (6)	
H19	0.2509	0.7270	0.0862	0.048*	
C15	0.19185 (13)	0.5876 (2)	0.18284 (14)	0.0429 (6)	
H15	0.1673	0.5897	0.2174	0.052*	
C6	0.24333 (13)	1.19804 (19)	0.14358 (14)	0.0416 (6)	
C18	0.26109 (12)	0.58116 (18)	0.08291 (12)	0.0367 (5)	
C9	0.38638 (14)	1.3271 (2)	−0.00520 (16)	0.0532 (7)	
H9	0.3651	1.3608	0.0253	0.064*	
C7	0.33364 (14)	1.1743 (2)	0.01789 (15)	0.0502 (7)	
H7	0.3256	1.1073	0.0076	0.060*	
C4	0.20356 (12)	1.13009 (19)	0.17502 (13)	0.0380 (6)	
C17	0.24587 (13)	0.49446 (19)	0.11196 (14)	0.0449 (6)	
H17	0.2594	0.4336	0.0971	0.054*	
C16	0.21082 (14)	0.4979 (2)	0.16270 (14)	0.0486 (7)	
H16	0.1999	0.4396	0.1834	0.058*	
C1	0.13502 (14)	1.0108 (2)	0.24051 (14)	0.0507 (7)	
H1	0.1108	0.9695	0.2634	0.061*	
C3	0.16615 (14)	1.1703 (2)	0.21661 (15)	0.0482 (7)	
H3	0.1647	1.2390	0.2226	0.058*	
C5	0.20473 (13)	1.02969 (19)	0.16908 (13)	0.0410 (6)	
H5	0.2304	1.0018	0.1408	0.049*	
C13	0.40755 (16)	1.1802 (2)	−0.06192 (17)	0.0606 (8)	
H13	0.4006	1.1126	−0.0708	0.073*	
C11	0.45847 (15)	1.3286 (3)	−0.08112 (17)	0.0575 (8)	
C8	0.37610 (13)	1.2281 (2)	−0.01660 (14)	0.0485 (7)	
C14	0.50138 (17)	1.3823 (3)	−0.1134 (2)	0.0713 (10)	
C2	0.13130 (14)	1.1097 (2)	0.24891 (16)	0.0535 (8)	
H2	0.1048	1.1361	0.2769	0.064*	
C10	0.42664 (15)	1.3771 (2)	−0.03712 (17)	0.0576 (8)	
H10	0.4328	1.4450	−0.0291	0.069*	
N3	0.26781 (11)	1.16107 (18)	0.09093 (12)	0.0452 (5)	
N2	0.20658 (11)	0.67179 (15)	0.15560 (11)	0.0417 (5)	
N4	0.30732 (11)	1.21732 (18)	0.06194 (12)	0.0474 (6)	
N1	0.17155 (11)	0.96955 (16)	0.20120 (11)	0.0443 (5)	
N5	0.53463 (17)	1.4260 (3)	−0.1381 (2)	0.1005 (13)	
P1	0.37466 (4)	0.89969 (6)	0.14663 (5)	0.0599 (2)	
O2	0.29542 (10)	0.50513 (15)	−0.01052 (10)	0.0563 (5)	
O1	0.25284 (10)	1.28063 (15)	0.16533 (11)	0.0584 (6)	
C20	0.29779 (12)	0.57604 (19)	0.02673 (13)	0.0409 (6)	
F5	0.38656 (13)	0.78661 (16)	0.13688 (14)	0.1030 (8)	
F6	0.35972 (14)	1.01041 (16)	0.15169 (18)	0.1254 (11)	
F1	0.3229 (3)	0.8764 (3)	0.1891 (3)	0.1111 (19)	0.773 (8)
F2	0.4206 (3)	0.9026 (5)	0.2114 (4)	0.182 (3)	0.773 (8)
F3	0.4237 (2)	0.9205 (3)	0.1009 (4)	0.129 (2)	0.773 (8)
F4	0.3249 (2)	0.8894 (4)	0.0790 (2)	0.1269 (19)	0.773 (8)
F1'	0.3816 (8)	0.8806 (8)	0.2243 (5)	0.096 (5)	0.227 (8)
F2'	0.4440 (4)	0.9236 (7)	0.1569 (9)	0.082 (4)	0.227 (8)
F3'	0.3702 (9)	0.9217 (9)	0.0687 (5)	0.112 (5)	0.227 (8)
F4'	0.3055 (4)	0.8774 (9)	0.1384 (11)	0.109 (5)	0.227 (8)
H28	0.2574 (13)	1.1069 (16)	0.0696 (14)	0.050 (9)*	
H29	0.3373 (14)	0.7020 (18)	0.0501 (13)	0.048 (9)*	

### Atomic displacement parameters (Å^2^)

**Table:**

	*U*^11^	*U*^22^	*U*^33^	*U*^12^	*U*^13^	*U*^23^
N8	0.0642 (18)	0.082 (2)	0.0669 (17)	−0.0083 (15)	0.0370 (15)	−0.0052 (15)
C28	0.0488 (17)	0.067 (2)	0.0496 (16)	−0.0054 (15)	0.0193 (14)	−0.0023 (15)
C25	0.0400 (15)	0.0643 (19)	0.0425 (15)	−0.0035 (13)	0.0168 (12)	−0.0011 (13)
C24	0.0556 (18)	0.0592 (19)	0.0508 (16)	0.0032 (14)	0.0264 (14)	0.0007 (14)
C26	0.060 (2)	0.0591 (19)	0.068 (2)	−0.0163 (15)	0.0323 (17)	−0.0065 (16)
C27	0.061 (2)	0.057 (2)	0.070 (2)	−0.0081 (15)	0.0337 (17)	−0.0143 (16)
C23	0.0533 (18)	0.0538 (17)	0.0513 (16)	−0.0046 (13)	0.0241 (14)	0.0040 (13)
C22	0.0434 (16)	0.0615 (19)	0.0422 (14)	−0.0043 (13)	0.0185 (13)	0.0001 (13)
C21	0.0528 (18)	0.0588 (19)	0.0508 (16)	−0.0022 (14)	0.0266 (14)	−0.0057 (14)
N7	0.0492 (14)	0.0615 (15)	0.0438 (13)	−0.0014 (12)	0.0254 (11)	−0.0007 (11)
N6	0.0535 (15)	0.0522 (15)	0.0478 (13)	−0.0051 (11)	0.0304 (12)	−0.0071 (11)
Ag1	0.07322 (19)	0.03326 (13)	0.07451 (18)	0.00004 (10)	0.03887 (14)	−0.00591 (10)
C12	0.064 (2)	0.075 (2)	0.062 (2)	0.0135 (18)	0.0322 (17)	0.0016 (18)
C19	0.0487 (16)	0.0332 (13)	0.0431 (14)	−0.0006 (11)	0.0210 (12)	0.0034 (11)
C15	0.0494 (16)	0.0379 (14)	0.0468 (14)	−0.0020 (11)	0.0230 (13)	−0.0003 (11)
C6	0.0461 (15)	0.0332 (14)	0.0469 (15)	0.0052 (11)	0.0122 (12)	0.0029 (11)
C18	0.0411 (14)	0.0351 (13)	0.0366 (13)	0.0005 (10)	0.0145 (11)	−0.0020 (10)
C9	0.0540 (18)	0.0563 (19)	0.0534 (17)	0.0099 (14)	0.0210 (14)	0.0056 (14)
C7	0.0539 (18)	0.0480 (17)	0.0508 (16)	−0.0006 (13)	0.0151 (14)	0.0028 (13)
C4	0.0431 (15)	0.0345 (13)	0.0377 (13)	0.0032 (11)	0.0107 (11)	−0.0017 (10)
C17	0.0579 (18)	0.0308 (13)	0.0502 (15)	0.0018 (12)	0.0210 (13)	−0.0012 (11)
C16	0.0652 (19)	0.0339 (14)	0.0519 (16)	−0.0052 (13)	0.0246 (14)	0.0049 (12)
C1	0.0609 (19)	0.0464 (16)	0.0517 (16)	−0.0040 (13)	0.0294 (15)	−0.0037 (13)
C3	0.0550 (17)	0.0375 (15)	0.0556 (16)	0.0041 (12)	0.0194 (14)	−0.0086 (12)
C5	0.0520 (16)	0.0349 (14)	0.0396 (13)	0.0029 (11)	0.0176 (12)	−0.0028 (11)
C13	0.069 (2)	0.054 (2)	0.063 (2)	0.0052 (15)	0.0251 (17)	−0.0041 (15)
C11	0.0524 (18)	0.067 (2)	0.0578 (18)	0.0096 (15)	0.0222 (15)	0.0139 (16)
C8	0.0455 (16)	0.0573 (18)	0.0444 (15)	0.0042 (13)	0.0123 (13)	0.0069 (13)
C14	0.072 (2)	0.070 (2)	0.082 (2)	0.0147 (18)	0.041 (2)	0.0162 (19)
C2	0.0587 (19)	0.0492 (17)	0.0604 (18)	0.0035 (14)	0.0321 (15)	−0.0082 (14)
C10	0.0587 (19)	0.0539 (19)	0.0648 (19)	0.0017 (14)	0.0238 (16)	0.0081 (15)
N3	0.0534 (15)	0.0370 (13)	0.0491 (13)	−0.0059 (10)	0.0197 (11)	−0.0014 (10)
N2	0.0493 (13)	0.0349 (12)	0.0466 (12)	0.0005 (9)	0.0247 (11)	0.0001 (9)
N4	0.0512 (14)	0.0456 (13)	0.0482 (13)	−0.0036 (11)	0.0163 (11)	0.0063 (11)
N1	0.0579 (15)	0.0331 (12)	0.0463 (12)	−0.0004 (10)	0.0215 (11)	−0.0037 (9)
N5	0.105 (3)	0.084 (3)	0.134 (3)	0.009 (2)	0.083 (3)	0.021 (2)
P1	0.0625 (5)	0.0379 (4)	0.0841 (6)	0.0022 (4)	0.0261 (5)	−0.0005 (4)
O2	0.0716 (14)	0.0488 (12)	0.0551 (12)	0.0005 (10)	0.0296 (11)	−0.0130 (10)
O1	0.0766 (15)	0.0328 (10)	0.0723 (14)	−0.0048 (10)	0.0310 (12)	−0.0021 (10)
C20	0.0463 (15)	0.0397 (14)	0.0403 (14)	0.0051 (11)	0.0179 (12)	0.0000 (11)
F5	0.139 (2)	0.0483 (12)	0.125 (2)	0.0164 (13)	0.0331 (17)	−0.0072 (13)
F6	0.142 (2)	0.0447 (13)	0.208 (3)	0.0124 (13)	0.083 (2)	0.0042 (16)
F1	0.141 (4)	0.087 (2)	0.128 (4)	0.010 (2)	0.085 (3)	0.026 (2)
F2	0.143 (5)	0.219 (6)	0.156 (5)	0.022 (4)	−0.051 (4)	−0.062 (4)
F3	0.112 (4)	0.094 (3)	0.207 (6)	−0.018 (2)	0.105 (4)	−0.012 (3)
F4	0.110 (3)	0.157 (4)	0.101 (3)	0.006 (3)	−0.017 (3)	0.017 (3)
F1'	0.149 (10)	0.085 (7)	0.064 (6)	−0.010 (7)	0.041 (7)	0.010 (5)
F2'	0.056 (5)	0.065 (6)	0.125 (9)	−0.004 (4)	0.020 (6)	−0.001 (6)
F3'	0.146 (11)	0.099 (8)	0.085 (7)	0.012 (8)	0.000 (7)	0.021 (6)
F4'	0.075 (7)	0.097 (8)	0.156 (11)	−0.012 (5)	0.020 (7)	−0.009 (8)

### Geometric parameters (Å, °)

**Table:**

N8—C28	1.134 (4)	C9—C8	1.387 (4)
N8—Ag1^i^	2.456 (3)	C9—H9	0.9500
C28—C25	1.440 (4)	C7—N4	1.279 (4)
C25—C26	1.378 (4)	C7—C8	1.461 (4)
C25—C24	1.389 (4)	C7—H7	0.9500
C24—C23	1.372 (4)	C4—C5	1.381 (4)
C24—H24	0.9500	C4—C3	1.386 (4)
C26—C27	1.384 (4)	C17—C16	1.379 (4)
C26—H26	0.9500	C17—H17	0.9500
C27—C22	1.379 (4)	C16—H16	0.9500
C27—H27	0.9500	C1—N1	1.347 (3)
C23—C22	1.390 (4)	C1—C2	1.369 (4)
C23—H23	0.9500	C1—H1	0.9500
C22—C21	1.465 (4)	C3—C2	1.370 (4)
C21—N7	1.271 (4)	C3—H3	0.9500
C21—H21	0.9500	C5—N1	1.341 (3)
N7—N6	1.382 (3)	C5—H5	0.9500
N6—C20	1.351 (4)	C13—C8	1.397 (4)
N6—H29	0.853 (18)	C13—H13	0.9500
Ag1—N1	2.172 (2)	C11—C10	1.387 (4)
Ag1—N2	2.199 (2)	C11—C14	1.443 (5)
Ag1—N8^ii^	2.456 (3)	C14—N5	1.130 (4)
C12—C13	1.384 (5)	C2—H2	0.9500
C12—C11	1.387 (5)	C10—H10	0.9500
C12—H12	0.9500	N3—N4	1.371 (3)
C19—N2	1.343 (3)	N3—H28	0.865 (18)
C19—C18	1.377 (3)	P1—F2	1.500 (5)
C19—H19	0.9500	P1—F1'	1.545 (9)
C15—N2	1.338 (3)	P1—F4'	1.555 (10)
C15—C16	1.381 (4)	P1—F6	1.559 (2)
C15—H15	0.9500	P1—F2'	1.560 (8)
C6—O1	1.216 (3)	P1—F3'	1.562 (9)
C6—N3	1.358 (3)	P1—F3	1.564 (4)
C6—C4	1.495 (4)	P1—F1	1.577 (3)
C18—C17	1.388 (4)	P1—F5	1.589 (2)
C18—C20	1.496 (3)	P1—F4	1.594 (4)
C9—C10	1.369 (4)	O2—C20	1.216 (3)
			
C28—N8—Ag1^i^	153.0 (3)	C12—C13—H13	119.6
N8—C28—C25	177.1 (4)	C8—C13—H13	119.6
C26—C25—C24	120.9 (3)	C10—C11—C12	120.1 (3)
C26—C25—C28	120.5 (3)	C10—C11—C14	119.2 (3)
C24—C25—C28	118.5 (3)	C12—C11—C14	120.7 (3)
C23—C24—C25	119.7 (3)	C9—C8—C13	118.5 (3)
C23—C24—H24	120.1	C9—C8—C7	121.3 (3)
C25—C24—H24	120.1	C13—C8—C7	120.3 (3)
C25—C26—C27	118.5 (3)	N5—C14—C11	178.6 (4)
C25—C26—H26	120.7	C1—C2—C3	119.4 (3)
C27—C26—H26	120.7	C1—C2—H2	120.3
C22—C27—C26	121.4 (3)	C3—C2—H2	120.3
C22—C27—H27	119.3	C9—C10—C11	120.1 (3)
C26—C27—H27	119.3	C9—C10—H10	119.9
C24—C23—C22	120.2 (3)	C11—C10—H10	119.9
C24—C23—H23	119.9	C6—N3—N4	119.2 (2)
C22—C23—H23	119.9	C6—N3—H28	126 (2)
C27—C22—C23	119.2 (3)	N4—N3—H28	114 (2)
C27—C22—C21	119.7 (3)	C15—N2—C19	117.9 (2)
C23—C22—C21	121.0 (3)	C15—N2—Ag1	120.36 (17)
N7—C21—C22	120.4 (3)	C19—N2—Ag1	121.45 (17)
N7—C21—H21	119.8	C7—N4—N3	115.7 (2)
C22—C21—H21	119.8	C5—N1—C1	117.3 (2)
C21—N7—N6	114.8 (3)	C5—N1—Ag1	121.31 (17)
C20—N6—N7	119.3 (2)	C1—N1—Ag1	121.18 (18)
C20—N6—H29	123 (2)	F2—P1—F4'	127.9 (7)
N7—N6—H29	116 (2)	F1'—P1—F4'	89.8 (7)
N1—Ag1—N2	156.22 (8)	F2—P1—F6	92.4 (3)
N1—Ag1—N8^ii^	109.53 (9)	F1'—P1—F6	94.9 (5)
N2—Ag1—N8^ii^	92.22 (9)	F4'—P1—F6	88.7 (5)
C13—C12—C11	119.3 (3)	F2—P1—F2'	50.8 (5)
C13—C12—H12	120.3	F1'—P1—F2'	88.9 (7)
C11—C12—H12	120.3	F4'—P1—F2'	178.3 (8)
N2—C19—C18	123.0 (2)	F6—P1—F2'	90.3 (4)
N2—C19—H19	118.5	F2—P1—F3'	139.5 (7)
C18—C19—H19	118.5	F1'—P1—F3'	177.5 (8)
N2—C15—C16	122.7 (2)	F4'—P1—F3'	92.5 (8)
N2—C15—H15	118.7	F6—P1—F3'	84.2 (4)
C16—C15—H15	118.7	F2'—P1—F3'	88.8 (7)
O1—C6—N3	123.4 (3)	F2—P1—F3	92.8 (4)
O1—C6—C4	120.7 (2)	F1'—P1—F3	130.6 (6)
N3—C6—C4	115.9 (2)	F4'—P1—F3	139.2 (7)
C19—C18—C17	118.5 (2)	F6—P1—F3	92.24 (19)
C19—C18—C20	123.1 (2)	F3'—P1—F3	47.2 (6)
C17—C18—C20	118.4 (2)	F2—P1—F1	90.2 (4)
C10—C9—C8	121.1 (3)	F1'—P1—F1	52.3 (6)
C10—C9—H9	119.5	F6—P1—F1	88.72 (18)
C8—C9—H9	119.5	F2'—P1—F1	140.8 (6)
N4—C7—C8	120.0 (3)	F3'—P1—F1	129.9 (7)
N4—C7—H7	120.0	F3—P1—F1	176.8 (3)
C8—C7—H7	120.0	F2—P1—F5	91.5 (3)
C5—C4—C3	117.9 (2)	F1'—P1—F5	88.1 (4)
C5—C4—C6	124.2 (2)	F4'—P1—F5	88.8 (5)
C3—C4—C6	117.7 (2)	F6—P1—F5	176.14 (19)
C16—C17—C18	119.0 (2)	F2'—P1—F5	92.2 (4)
C16—C17—H17	120.5	F3'—P1—F5	92.9 (4)
C18—C17—H17	120.5	F3—P1—F5	87.63 (19)
C17—C16—C15	118.9 (2)	F1—P1—F5	91.21 (18)
C17—C16—H16	120.5	F2—P1—F4	176.3 (4)
C15—C16—H16	120.5	F1'—P1—F4	139.5 (6)
N1—C1—C2	122.8 (3)	F4'—P1—F4	50.2 (7)
N1—C1—H1	118.6	F6—P1—F4	90.8 (2)
C2—C1—H1	118.6	F2'—P1—F4	131.2 (6)
C2—C3—C4	119.2 (3)	F3—P1—F4	89.0 (3)
C2—C3—H3	120.4	F1—P1—F4	87.9 (3)
C4—C3—H3	120.4	F5—P1—F4	85.3 (2)
N1—C5—C4	123.4 (2)	O2—C20—N6	123.8 (2)
N1—C5—H5	118.3	O2—C20—C18	121.3 (2)
C4—C5—H5	118.3	N6—C20—C18	114.9 (2)
C12—C13—C8	120.9 (3)		
			
C26—C25—C24—C23	−0.5 (5)	C12—C13—C8—C7	−179.0 (3)
C28—C25—C24—C23	178.8 (3)	N4—C7—C8—C9	−3.3 (5)
C24—C25—C26—C27	1.2 (5)	N4—C7—C8—C13	176.4 (3)
C28—C25—C26—C27	−178.1 (3)	N1—C1—C2—C3	−0.4 (5)
C25—C26—C27—C22	−1.0 (5)	C4—C3—C2—C1	1.2 (5)
C25—C24—C23—C22	−0.4 (5)	C8—C9—C10—C11	−0.7 (5)
C26—C27—C22—C23	0.2 (5)	C12—C11—C10—C9	1.4 (5)
C26—C27—C22—C21	177.6 (3)	C14—C11—C10—C9	−179.0 (3)
C24—C23—C22—C27	0.6 (5)	O1—C6—N3—N4	2.7 (4)
C24—C23—C22—C21	−176.8 (3)	C4—C6—N3—N4	−176.6 (2)
C27—C22—C21—N7	169.9 (3)	C16—C15—N2—C19	0.9 (4)
C23—C22—C21—N7	−12.7 (5)	C16—C15—N2—Ag1	−173.5 (2)
C22—C21—N7—N6	177.8 (3)	C18—C19—N2—C15	−0.6 (4)
C21—N7—N6—C20	179.8 (3)	C18—C19—N2—Ag1	173.8 (2)
N2—C19—C18—C17	0.1 (4)	N1—Ag1—N2—C15	−169.9 (2)
N2—C19—C18—C20	178.6 (3)	N8^ii^—Ag1—N2—C15	−13.3 (2)
O1—C6—C4—C5	−159.2 (3)	N1—Ag1—N2—C19	15.9 (4)
N3—C6—C4—C5	20.1 (4)	N8^ii^—Ag1—N2—C19	172.5 (2)
O1—C6—C4—C3	16.2 (4)	C8—C7—N4—N3	179.3 (3)
N3—C6—C4—C3	−164.5 (3)	C6—N3—N4—C7	172.6 (3)
C19—C18—C17—C16	0.1 (4)	C4—C5—N1—C1	0.7 (4)
C20—C18—C17—C16	−178.5 (3)	C4—C5—N1—Ag1	−173.5 (2)
C18—C17—C16—C15	0.2 (4)	C2—C1—N1—C5	−0.6 (4)
N2—C15—C16—C17	−0.7 (5)	C2—C1—N1—Ag1	173.6 (2)
C5—C4—C3—C2	−1.1 (4)	N2—Ag1—N1—C5	−25.6 (4)
C6—C4—C3—C2	−176.9 (3)	N8^ii^—Ag1—N1—C5	179.3 (2)
C3—C4—C5—N1	0.2 (4)	N2—Ag1—N1—C1	160.4 (2)
C6—C4—C5—N1	175.6 (3)	N8^ii^—Ag1—N1—C1	5.3 (2)
C11—C12—C13—C8	−0.1 (5)	N7—N6—C20—O2	−3.9 (4)
C13—C12—C11—C10	−1.0 (5)	N7—N6—C20—C18	176.5 (2)
C13—C12—C11—C14	179.5 (3)	C19—C18—C20—O2	−150.7 (3)
C10—C9—C8—C13	−0.4 (5)	C17—C18—C20—O2	27.8 (4)
C10—C9—C8—C7	179.4 (3)	C19—C18—C20—N6	29.0 (4)
C12—C13—C8—C9	0.8 (5)	C17—C18—C20—N6	−152.5 (3)

Symmetry codes: (i) *x*+1/2, −*y*+3/2, *z*−1/2; (ii) *x*−1/2, −*y*+3/2, *z*+1/2.

### Hydrogen-bond geometry (Å, °)

**Table:**

*D*—H···*A*	*D*—H	H···*A*	*D*···*A*	*D*—H···*A*
N3—H28···O2^iii^	0.865 (18)	2.15 (2)	2.990 (3)	162 (3)
N6—H29···F5	0.853 (18)	2.21 (2)	3.001 (4)	155 (3)

Symmetry codes: (iii) −*x*+1/2, −*y*+3/2, −*z*.
